# Tirzepatide cardiovascular event risk assessment: a pre-specified meta-analysis

**DOI:** 10.1038/s41591-022-01707-4

**Published:** 2022-02-24

**Authors:** Naveed Sattar, Darren K. McGuire, Imre Pavo, Govinda J. Weerakkody, Hiroshi Nishiyama, Russell J. Wiese, Sophia Zoungas

**Affiliations:** 1 Institute of Cardiovascular and Medical Sciences, BHF Glasgow Cardiovascular Research Centre, University of Glasgow, Glasgow, UK; 2 University of Texas Southwestern Medical Center and Parkland Health and Hospital System, Dallas, TX USA; 3 Eli Lilly Regional Operations GmbH, Vienna, Austria; 4 Eli Lilly and Company, Indianapolis, IN USA; 5 School of Public Health and Preventative Medicine, Monash University, Melbourne, VC Australia

**Keywords:** Diabetes complications, Cardiovascular diseases

## Abstract

Tirzepatide is a novel, once weekly, dual GIP/GLP-1 receptor agonist and is under development for the treatment of type 2 diabetes (T2D) and obesity. Its association with cardiovascular outcomes requires evaluation. This pre-specified cardiovascular meta-analysis included all seven randomized controlled trials with a duration of at least 26 weeks from the tirzepatide T2D clinical development program, SURPASS. The pre-specified primary objective of this meta-analysis was the comparison of the time to first occurrence of confirmed four-component major adverse cardiovascular events (MACE-4; cardiovascular death, myocardial infarction, stroke and hospitalized unstable angina) between pooled tirzepatide groups and control groups. A stratified Cox proportional hazards model, with treatment as a fixed effect and trial-level cardiovascular risk as the stratification factor, was used for the estimation of hazard ratios (HRs) and confidence intervals (CIs) comparing tirzepatide to control. Data from 4,887 participants treated with tirzepatide and 2,328 control participants were analyzed. Overall, 142 participants, 109 from the trial with high cardiovascular risk and 33 from the six trials with lower cardiovascular risk, had at least one MACE-4 event. The HRs comparing tirzepatide versus controls were 0.80 (95% CI, 0.57–1.11) for MACE-4; 0.90 (95% CI, 0.50–1.61) for cardiovascular death; and 0.80 (95% CI, 0.51–1.25) for all-cause death. No evidence of effect modifications was observed for any subgroups, although the evidence was stronger for participants with high cardiovascular risk. Tirzepatide did not increase the risk of major cardiovascular events in participants with T2D versus controls.

## Abstract (web summary)

Meta-analysis of cardiovascular secondary outcomes from the SURPASS program, testing efficacy of a new novel dual GIP/GLP-1 receptor agonist tirzepatide, demonstrates cardiovascular safety in patients with type 2 diabetes.

## Main

T2D is responsible for an average two-fold-higher risk for cardiovascular events, such as coronary heart disease, ischemic stroke, hospitalization for heart failure (HHF) and vascular death, independent of other established risk factors^1,2^.

Glucagon-like peptide-1 receptor agonists (GLP-1RAs) are now considered the first choice of injectable therapy for many people with T2D, with several members of the class having proven cardiovascular efficacy^3^. Building on that concept, the combined glucose-dependent insulinotropic polypeptide (GIP) and GLP-1RAs have been proposed as a novel therapeutic option for T2D^4^. Tirzepatide is one such molecule that has shown marked glycemia and weight benefits in a series of trials^5,6^. For example, when compared to placebo, semaglutide 1 mg per week, dulaglutide 1.5 mg per week and insulin degludec or insulin glargine 100 U ml^−1^, tirzepatide was more effective in reducing glycated hemoglobin (HbA1c) and weight in people with T2D over a 26−52-week treatment period^5,6^. Tirzepatide might improve glycemic control beyond that of GLP-1RAs through direct and indirect actions on the pancreas and other tissues, including enhancing pancreatic β-cell insulin secretion, reducing glucose-adjusted glucagon secretion and improving insulin sensitivity beyond the levels usually explained by weight loss^6–8^. Additionally, tirzepatide’s anorexigenic effect might exceed that of GLP-1RAs by integrating the activation signals of both GIP and GLP-1 receptor pathways in the brain^7,9^. Among other beneficial effects, tirzepatide is also associated with improvements in lipoprotein profiles (more than GLP-1RAs), blood pressure and several biomarkers of inflammation^7,10^. Notably, glycemic and weight effects appear to be maintained for at least 2 years while receiving tirzepatide, the longest observation period for this drug^11^.

Despite favorable effects of tirzepatide on a range of cardiovascular risk factors, to date its cardiovascular safety has been reported from results of only a single trial, SURPASS-4 (ref. ^11^). This trial, which compared tirzepatide treatment to insulin glargine 100 U ml^−1^ treatment in people with T2D at increased cardiovascular risk, suggested no significant difference in the incidence of major cardiovascular events.

As required by the US Food and Drug Administration (FDA) and the European Medicines Agency (EMA)^12–15^, and to extend the safety evaluation in a broader population with T2D, the tirzepatide clinical development program for treatment of T2D (SURPASS) was also designed to evaluate the drug’s cardiovascular safety in people with T2D at low, medium and high cardiovascular risk^12^. Therefore, a cardiovascular safety meta-analysis of data, from all phase 2 and phase 3 clinical trials with planned treatment duration of at least 26 weeks and at least one randomized comparator arm among adults with T2D, was conducted to assess the safety of tirzepatide with regard to major cardiovascular events relative to various randomized comparators.

Here we present the results of these pre-specified cardiovascular safety meta-analyses and selected post hoc exploratory analyses of interest, based on prospectively collected and centrally adjudicated MACE events.

## Results

Data from one phase 2 trial, five international phase 3 trials and one regional phase 3 trial in Japan, each with at least one MACE-4 event, were included in this meta-analysis (Supplementary Table 1).

### Baseline demographics and clinical characteristics

A total of 7,215 randomized participants were included in the meta-analysis (pooled tirzepatide group treated with a mean assigned dose of 9.9 mg per week of tirzepatide, *n* = 4,887 (1 mg, *n* = 52; 5 mg, *n* = 1,608; 10 mg, *n* = 1,606; 15 mg, *n* = 1621) and pooled comparator group: *n* = 2,328 (placebo, *n* = 286; insulin degludec, *n* = 360; insulin glargine, *n* = 1,000, semaglutide 1 mg, *n* = 469; dulaglutide 1.5 mg, *n* = 54; dulaglutide 0.75 mg, *n* = 159) (Table 1). The total study drug exposure was 4,404.3 patient-years in the pooled tirzepatide group and 2,470.7 patient-years in the pooled comparator group, wherein participants were exposed for a median duration of 44 weeks. The total duration of follow-up was 5,100.9 patient-years in the pooled tirzepatide group and 2,757.2 patient-years in the pooled comparator group, as the participants were followed-up for a median duration of 55.3 weeks. Premature trial discontinuations were reported in 321 (6.7%) participants in the pooled tirzepatide group compared to 208 (8.2%) participants in the pooled comparator group. Overall, the most frequently reported reasons for premature discontinuation from the study were similar across both pooled treatment groups and included withdrawal by participant (pooled tirzepatide group: 117 (2.3%) participants as compared to the pooled comparator group: 70 (2.8%) participants) and lost to follow-up (pooled tirzepatide group: 73 (1.5%) participants as compared to the pooled comparator group: 47 (2.0%) participants).

**Table 1:** Baseline demographics and clinical characteristics

Parameter	All tirzepatide *N* = 4,887	All comparator *N* = 2,328	Total *N* = 7,215
Age, years	58.7 (9.9)	59.0 (10.0)	58.8 (9.9)
Age category, *n* (%)			
<65 years	3,421 (68.5)	1,496 (67.7)	4,917 (68.1)
≥65 years	1,466 (31.5)	832 (32.3)	2,298 (31.9)
Sex, female, *n* (%)	2,163 (43.8)	962 (42.6)	3,125 (43.3)
Ethnicity, *n* (%)			
Hispanic or Latino	2,039 (42.3)	1,020 (42.8)	3,059 (42.4)
Not Hispanic or Latino	2,212 (45.8)	1,091 (45.6)	3,303 (45.8)
Not reported	636 (11.9)	217 (11.7)	853 (11.8)
Race, *n* (%)			
American Indian or Alaska Native	356 (7.4)	164 (6.7)	520 (7.2)
Asian	792 (15.1)	273 (14.1)	1,065 (14.8)
Black or African American	185 (3.8)	69 (2.9)	254 (3.5)
Native Hawaiian or Other Pacific Islander or Multiple	44 (1.0)	36 (1.3)	80 (1.1)
White	3,507 (72.6)	1,783 (75.1)	5,290 (73.4)
Country, *n* (%)			
United States	1,084 (21.7)	503 (22.9)	1,587 (22.0)
Outside United States	3,803 (78.3)	1,825 (77.1)	5,628 (78.0)
Weight, kg	91.12 (20.32)	90.59 (20.36)	90.98 (20.37)
BMI, kg m^−^^2^	32.849 (6.263)	32.673 (6.259)	32.791 (6.262)
Duration of diabetes, years	9.23 (6.81)	9.22 (6.78)	9.26 (6.83)
HbA1c, %	8.30 (0.94)	8.27 (0.91)	8.29 (0.93)
HbA1c category, *n* (%)			
≤8.5%	3,177 (64.2)	1475 (64.9)	4,652 (64.5)
>8.5%	1,709 (35.8)	853 (35.1)	2,562 (35.5)
Systolic blood pressure, mmHg	131.88 (14.41)	132.02 (14.58)	131.94 (14.49)
Diastolic blood pressure, mmHg	79.22 (9.28)	79.36 (9.40)	79.26 (9.34)
HDL cholesterol, mg dl^−1^	44.60 (11.81)	44.86 (11.52)	44.67 (11.73)
LDL cholesterol, mg dl^−1^	93.07 (34.49)	94.28 (34.85)	93.45 (34.63)
Triglycerides, mg dl^−1^	185.67 (131.72)	181.61 (134.30)	184.53 (133.16)
Total cholesterol, mg dl^−1^	173.18 (41.76)	173.90 (42.02)	173.40 (41.88)
Smoking history, yes, *n* (%)	1,453 (30.7)	719 (28.9)	2,172 (30.1)
Current smoking status, yes, *n* (%)	822 (16.6)	416 (18.5)	1238 (17.2)
UACR, mg g^−1^, median	11.0	12.0	12.0
UACR category, *n* (%)			
Microalbuminuria	1,156 (24.2)	560 (23.4)	1,716 (24.0)
Macroalbuminuria	258 (5.6)	123 (4.6)	381 (5.3)
eGFR, ml/min/1.73 m^2^	89.02 (18.88)	88.99 (18.60)	89.04 (18.76)
eGFR category, *n* (%)			
<60 ml/min/1.73 m^2^	363 (8.4)	233 (8.2)	596 (8.3)
≥60 ml/min/1.73 m^2^	4,523 (91.6)	2,094 (91.8)	6,617 (91.7)

Data are presented as mean (s.d.) unless otherwise indicated (mITT population). Percentage is based on the number of participants with non-missing measurement at baseline. Data are strata size adjusted estimate. Strata are defined as trial-level cardiovascular risk (SURPASS-4 forms one stratum, and all other trials form one stratum). eGFR, estimated glomerular filtration rate; HDL, high-density lipoprotein; LDL, low-density lipoprotein; *N*, number of participants in the population; *n,* number of participants in the specified category; UACR, urine albumin to creatinine ratio.

The baseline demographic characteristics were balanced across the pooled tirzepatide and pooled comparator group (Table 1). Overall, participants had a mean baseline HbA1c of 8.3%, a mean diabetes duration of 9.3 years and a mean body mass index (BMI) of 32.8 kg m^−2^. The most frequently reported cardiovascular risk factors were hypertension and dyslipidemia (4,627 (73.9%) participants had hypertension and 4,237 (67.7%) participants had dyslipidemia) (Supplementary Table 2). In addition, 2,187 (34.9%) participants had a history of cardiovascular disease. No clinically relevant differences were observed across the pooled treatment groups in the cardiovascular risk factors at baseline. At trial level, the major indices for high cardiovascular risk were markedly different. Notably, history of cardiovascular disease was present in 86.9% of participants from SURPASS-4, whereas the prevalence of such conditions in the other trials was 5.4–18.3% (Supplementary Table 3).

### Cardiovascular safety meta-analysis

**Fig. 1:**
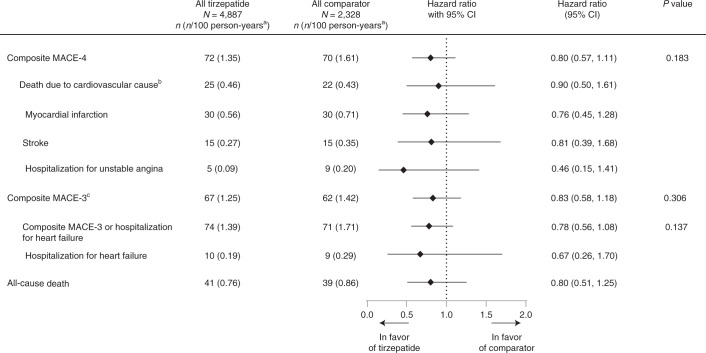
Primary and secondary cardiovascular outcomes confirmed by central-blinded adjudication. Data are point estimates of HR (illustrated by the diamond symbol) and range of two-sided 95% CI of the HR. ^a^Strata size adjusted estimate. Strata are defined as trial-level cardiovascular risk (SURPASS-4 forms one stratum, and all other trials form one stratum) ^b^Death due to cardiovascular cause includes adjudication-confirmed death due to cardiovascular or undetermined cause. ^c^MACE-3 includes death due to cardiovascular or undetermined cause, MI and stroke. Note: *P* values were based on the Wald chi-square test. *n*, number of participants in the specified category.

**Fig. 2:**
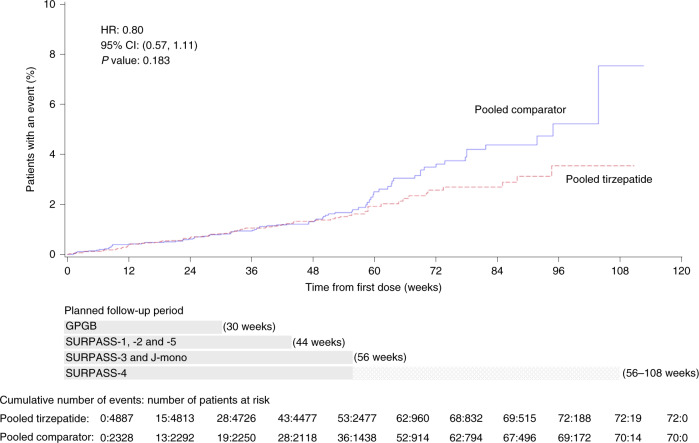
Adjusted Kaplan–Meier plot of pooled tirzepatide versus pooled comparator effect on time to first occurrence of adjudication-confirmed MACE-4 (primary outcome). Gray bars represent the planned follow-up period for trials GPGB (30 weeks); SURPASS-1, SURPASS-2 and SURPASS-5 (44 weeks); SURPASS-3 and SURPASS J-mono (56 weeks); and SURPASS-4 (56–108 weeks). Note: *P* values were based on the Wald chi-square test.

**Extended Data Fig. 1:**
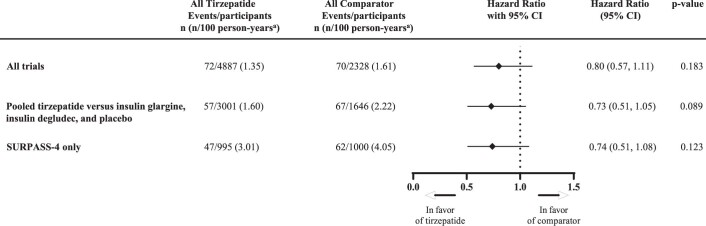
Time-to-event analyses of composite adjudication-confirmed MACE-4. Data are point estimate of HR (illustrated by the diamond symbol) and range of 2-sided 95% CI of the HR. ^a^Strata size adjusted estimate. Strata are defined as trial-level cardiovascular risk (SURPASS-4 forms one stratum, and all other trials form one stratum). Abbreviations: CI = confidence interval; HR = hazard ratio; iDeg = insulin degludec; iGlar = insulin glargine; MACE = major adverse cardiovascular events; n = number of participants in the specified category; PBO = placebo; TZP = tirzepatide.

Results from time-to-event analysis of composite MACE-4 and individual components for the composite outcome included 142 participants with at least one MACE-4 (47 participants with cardiovascular deaths, 60 participants with myocardial infarction (MI) events, 30 participants with stroke events and 14 participants with hospitalized unstable angina (HUA)) and are presented in Fig. 1. Tirzepatide treatment was not associated with increased risk of the MACE-4 outcome (HR of 0.80 (95% CI, 0.57–1.11)) (Fig. 2 and Extended Data Fig. 1), cardiovascular death (HR = 0.90 (95% CI, 0.50–1.61)), MI (HR = 0.76 (95% CI, 0.45–1.28)), stroke (HR = 0.81 (95% CI, 0.39–1.68)) and HUA (HR = 0.46 (95% CI, 0.15–1.41)). The rate of MACE-4 was markedly different among the individual trials in line with the differing cardiovascular risk profiles of trial participants. The highest rate was observed in SURPASS-4, 3.52 per 100 patient-years, with lower rates observed in the other trials, 0.26–1.13 per 100 patient-years (Supplementary Table 4).

The results of the comparisons among the treatment groups for the MACE-3 outcomes demonstrated an HR of 0.83 (95% CI, 0.58–1.18) for MACE-3; an HR of 0.78 (95% CI, 0.56–1.08) for composite outcome of MACE-3 or HHF; and an HR of 0.67 (95% CI, 0.26–1.70) for HHF. All-cause death had an HR of 0.80 (95% CI, 0.51–1.25) (Fig. 1).

**Fig. 3:**
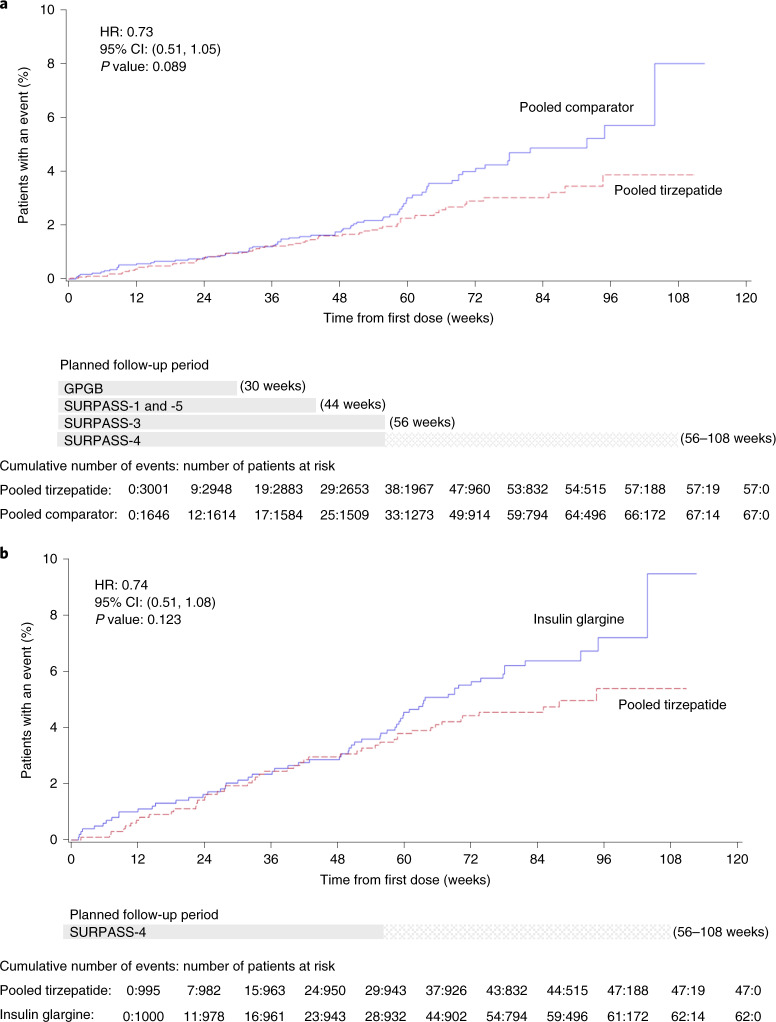
Time to first occurrence of adjudication-confirmed MACE-4 by trial groupings. Adjusted Kaplan–Meier estimates. **a**, Time to first occurrence of composite MACE-4, pooled tirzepatide versus insulin glargine, insulin degludec and placebo combined and mITT population. Gray bars represent the planned follow-up period for trials GPGB (30 weeks); SURPASS-1 and SURPASS-5 (44 weeks); and SURPASS-3 (56 weeks). **b**, Time to first occurrence of adjudicated-confirmed MACE-4, pooled tirzepatide versus insulin glargine, SURPASS-4 only and mITT population. Gray bar represents the planned follow-up period for SURPASS-4 (56–108 weeks). Note: *P* values were based on the Wald chi-square test.

Time to first occurrence of confirmed MACE-4 for trials including tirzepatide versus insulin glargine, insulin degludec and placebo combined demonstrated an HR of 0.73 (95% CI, 0.51–1.05) (*P* = 0.089) (Fig. 3a and Extended Data Fig. 1). Time to first occurrence of confirmed MACE-4 for SURPASS-4 only (tirzepatide versus insulin glargine in a high-cardiovascular-risk population) demonstrated an HR of 0.74 (95% CI, 0.51–1.08) (*P* = 0.123) (Extended Data Fig. 1).

**Fig. 4:**
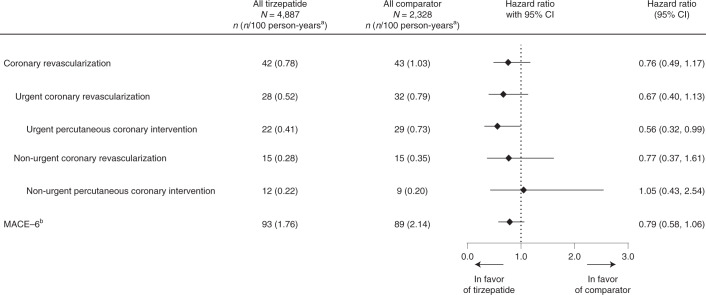
Other adjudication-confirmed cardiovascular outcomes. Data are point estimates of HR (illustrated by the diamond symbol) and range of two-sided 95% CI of the HR. ^a^Strata size adjusted estimate. Strata are defined as trial-level cardiovascular risk (SURPASS-4 forms one stratum, and all other trials form one stratum). ^b^MACE-6 includes MACE-3 and all other adjudication-confirmed cardiovascular outcomes: HUA, HHF and coronary revascularization. *n*, number of participants in the specified category.

In the trials included in this meta-analysis, 85 participants underwent at least one confirmed coronary revascularization procedure during the study: 60 participants (ten surgical and 51 percutaneous) were classified as undergoing an urgent procedure, and 30 participants (ten surgical and 21 percutaneous) were classified as undergoing a non-urgent procedure. Additionally, 182 participants suffered a first occurrence of MACE-6—the composite of MACE-4 plus HHF or coronary revascularizations. Post hoc analyses of the comparisons among the treatment groups for coronary revascularizations demonstrated an HR of 0.76 (95% CI, 0.49–1.17), including 0.67 (95% CI, 0.40–1.13) for urgent revascularizations and 0.77 (95% CI, 0.37–1.61) for non-urgent revascularizations. MACE-6 had an HR of 0.79 (95% CI, 0.58–1.06) (Fig. 4).

**Fig. 5:**
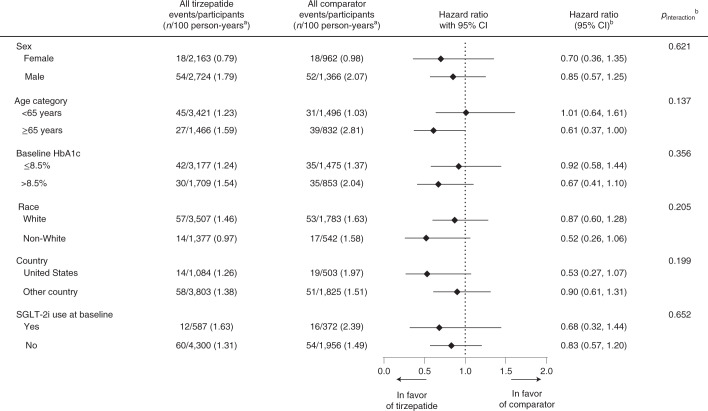
Subgroup analyses of the effects of tirzepatide on the adjudication-confirmed composite MACE-4. Data are point estimates of HR (illustrated by the diamond symbol) and range of two-sided 95% CI of the HR. ^a^Strata size adjusted estimate. Strata are defined as trial-level cardiovascular risk (SURPASS-4 forms one stratum, and all other trials form one stratum). ^b^Derived from a Cox proportional hazards model with treatment (pooled tirzepatide versus pooled comparator), the subgroup variable and the treatment-by-subgroup interaction term as fixed effects, stratified by study-level cardiovascular risk (SURPASS-4 forms one stratum, and all other trials form one stratum) for subgroups other than stratum. Pre-specified subgroup analyses were by sex, age and baseline HbA1c; the rest of the analyses were post hoc. *P* value is from the Wald chi-square test. *N*, number of participants in the subgroup in population; *n*, number of participants in the specified category.

Subgroup analyses for the primary outcome of MACE-4 by sex, age, baseline HbA1c, race, US or non-US clinical sites and baseline sodium glucose co-transporter 2 inhibitor (SGLT-2i) use demonstrated no significant effect modification (all *P*_interaction_ > 0.1) (Fig. 5).

Before the completion of SURPASS-4 and SURPASS J-mono, an interim analysis was performed for regulatory purposes. This analysis included 116 participants who had experienced at least one component of the MACE-4 composite endpoint. Once weekly tirzepatide and treatment exposure of up to 104 weeks (median follow-up of 55 weeks) resulted in an HR of 0.81 (97.85% CI, 0.52–1.26) for the pooled tirzepatide group compared to the pooled comparator group from 116 composite MACE-4 endpoints (pooled tirzepatide: 60 (1.37 per 100 person-years) and pooled comparator: 56 (1.60 per 100 person-years)) and, therefore, met the criteria that treatment with tirzepatide was not associated with excess cardiovascular risk and initiated early close-out of SURPASS-4.

## Discussion

This prospectively planned, pooled individual participant data, cardiovascular safety meta-analysis was conducted for the tirzepatide T2D clinical development program, SURPASS. The overall cardiovascular findings indicate that tirzepatide treatment given for a median duration of just over 1 year, at a mean randomization dose of 9.9 mg per week to a population with just over one-third having established cardiovascular disease, is not associated with increased cardiovascular risk when compared to placebo or comparators not known to be cardioprotective, with point estimates of HR < 1.0 and upper confidence limits of 95% CI < 1.8 for all MACE components. Furthermore, the incidence of MACE-4 with tirzepatide starts to diverge from the control groups after approximately 1 year. A similar pattern was observed in most previous GLP-1RA cardiovascular outcomes trials (CVOTs)^16–18^.

The population in the SURPASS clinical development program included a wide spectrum of people on the continuum of T2D—from participants treated with diet and exercise alone with a mean duration of diabetes of 4.7 years (SURPASS-1) to participants for whom basal insulin treatment was insufficient with a mean duration of diabetes of 13.3 years (SURPASS-5). Similarly, participants’ cardiovascular risk varied among trials. The SURPASS-4 trial enrolled participants at especially high cardiovascular risk, with enrichment criteria including previous cardiovascular event, established atherosclerotic cardiovascular disease and/or chronic kidney disease or heart failure. Consequently, as anticipated in the planning of the cardiovascular safety meta-analysis, this trial had the highest MACE incidence and contributed considerably to events analyzed in the present study. On the other hand, as similar changes in cardiovascular surrogate markers (HbA1c, weight, blood pressure and lipoproteins) by tirzepatide were observed in studies with high or moderate cardiovascular risk, it was assumed that the relative treatment effect would not be different regardless of the participants’ baseline cardiovascular risk level^6,11,19^. Moreover, baseline cardiovascular risk did not modify the estimate of treatment effects of GLP-1RAs on MACE^20^.

The comparators across the trials’ program were diverse. Some of the comparators, such as GLP-1RAs dulaglutide and semaglutide, have proven cardiovascular benefits as reflected in their product-labeled indications and endorsement across professional society guidelines and recommendations^16,17,21^, which would attenuate the comparative estimate of cardiovascular effect of tirzepatide. It was assumed, however, that any such effect would be negligible due to the short treatment duration of pooled trials and apparent lag time required to observe MACE benefits with dulaglutide or semaglutide (as reported by the placebo-controlled trials^16,22^). Placebo and the basal insulins included in the trials are considered to be neutral with regard to cardiovascular risk^18,21^, with pre-specified analyses of this subset of comparators likely to more accurately reflect the cardiovascular safety of tirzepatide. Both subset analyses, including an analysis of the SURPASS-4 trial only and an analysis of all insulin and placebo comparator trials, were generally consistent with the MACE-4 result of the entire pooled population. Collectively, these data meet the regulatory cardiovascular safety criterion for consideration of a novel anti-hyperglycemic medication for initial approval, statistically excluding the upper confidence limit of the 95% CI of 1.8. Furthermore, some of these findings provide optimism for the potential positive cardiovascular effects of tirzepatide (aiming for a maximum dose of 15 mg per day) being tested in the ongoing outcome trial SURPASS-CVOT (NCT04255433)^23^.

All individual trials had a balanced distribution of cardiovascular risk factors between pooled tirzepatide and control groups. However, this was not the case when participants were pooled from all trials. In SURPASS-4 (that is, participants with high cardiovascular risk), there was a higher ratio of participants in the control arm who had cardiovascular risk factors due to the 1:1 randomization scheme between the tirzepatide and insulin glargine groups. Therefore, these meta-analyses were based on pre-specified estimates adjusted for different randomization ratios between pooled tirzepatide versus pooled control in different trial-level cardiovascular risk strata. Without adjustment, the control group would be enriched with high-cardiovascular-risk participants, subject to more MACE, and would thus bias the results toward a lower HR in favor of tirzepatide.

In addition to the pre-specified cardiovascular analyses, post hoc analysis of the incidence of adjudication-confirmed coronary revascularizations was also assessed. The HRs overall, as well as in the sub-analysis stratified by urgent and non-urgent coronary revascularizations, were generally consistent with the primary and secondary cardiovascular safety findings. Moreover, the findings of the post hoc MACE-6 analysis, including first occurrence of all adjudication-confirmed cardiac events that yielded a larger number of events for analysis compared to MACE-4 or MACE-3, were broadly in line with the main cardiovascular findings.

The proportion of participants who were missing MACE assessments at the end of each trial was similar to contemporary glycemic trials and slightly higher than single CVOTs with MACE as the primary endpoints (3–4% at the maximum^16,22,24,25^). The missing assessments due to adverse events were balanced among pooled treatment groups; therefore, missing MACE assessments were considered missing at random, and the potential biases introduced due to missing data for the cardiovascular endpoint were likely minimal.

The present cardiovascular meta-analyses include a broad clinical population at different stages of their T2D course. The inclusion of a large number of participants with a history of multiple cardiovascular risk factors, prior events and prevalent cardiovascular disease, and participants with a trial treatment duration of up to 24 months, supported the assessment of cardiovascular safety^26–35^. Relatively few MACE were reported in participants with lower cardiovascular risk up to 56 weeks of follow-up despite high total investigational product exposures. Thus, cardiovascular safety assessment of tirzepatide was less robust in this subgroup.

Similarly to other clinical development programs of anti-hyperglycemic therapies for T2D, a limitation of these cardiovascular meta-analyses was the inclusion of tirzepatide clinical trials that excluded participants with recent unstable cardiovascular disease (for example, New York Heart Association class IV heart failure and recent cardiovascular events).

In conclusion, treatment with once weekly tirzepatide at the doses of 5 mg, 10 mg and 15 mg, with controlled treatment exposure of up to 104 weeks, was not associated with increased risk for cardiovascular events in people with T2D across a spectrum of T2D duration and cardiovascular risk levels.

## Methods

### Trials

This pre-specified meta-analysis of all randomized controlled trials of at least 26-weeks duration from the tirzepatide clinical development program included seven clinical trials: one phase 2 trial, five international phase 3 trials and one regional phase 3 trial in Japan^5–7,11,19,36,37^. Two trials were excluded: one phase 2 trial with 111 participants and 12-weeks duration and one uncontrolled phase 3 safety trial conducted in Japan. Trial durations ranged from 26 to 104 weeks (Supplementary Table 1). Individual data from participants randomized to tirzepatide (1 mg, 5 mg, 10 mg or 15 mg) and randomized to placebo or active comparator (insulin degludec, insulin glargine, semaglutide 1 mg or dulaglutide (1.5 mg or 0.75 mg)) were pooled.

### Participant population and randomization

The trials included individuals aged 18 years or older with T2D inadequately controlled with diet and exercise with or without metformin, with baseline HbA1c ranging from 7.0% to 10.5% and BMI of 23 kg m^−2^ or higher, depending on the trial. Notably, SURPASS-4 included a high-cardiovascular-risk population and was designed to contribute the majority (that is, approximately 80%) of the MACE-4 endpoints for evaluating cardiovascular safety, as compared to the other trials. Participants were randomly assigned 2:1 to tirzepatide (1 mg, 5 mg, 10 mg or 15 mg) or comparator (placebo or dulaglutide 1.5 mg) in the phase 2 trial; 3:1 to tirzepatide (5 mg, 10 mg or 15 mg) or comparators (placebo, semaglutide 1 mg, insulin degludec or dulaglutide 0.75 mg) in the phase 3 trials other than SURPASS-4; and 1:1 to tirzepatide (5 mg, 10 mg or 15 mg) or insulin glargine in SURPASS-4 (refs. ^5–7,11,19,36,37^).

Each trial was prospectively registered at ClinicalTrials.gov, received institutional review board approval for each participating center and was conducted in accordance with the Declaration of Helsinki. All participants provided written informed consent for trial participation.

### Cardiovascular event adjudication

MACE were prospectively captured and centrally adjudicated using similar event definitions across the trials’ program by personnel blinded to randomized assignment by the Duke Clinical Research Institute Clinical Endpoint Committee (CEC) (for the phase 2 trial) and the Cleveland Clinic Coordinating Center for Clinical Research CEC (for the phase 3 trials). The CEC reviewed all deaths (adjudicated cardiovascular and non-cardiovascular) and potential cardiovascular outcome events, including acute coronary syndromes (MI and HUA), coronary revascularization procedures (coronary artery bypass grafting and percutaneous coronary interventions), HHF and cerebrovascular events (stroke and transient ischemic attack).

### Outcomes

The primary outcome was time to first occurrence of CEC-confirmed MACE-4 (including cardiovascular death, MI, stroke and HUA). MACE-4 has been used in meta-analyses for several new diabetes medications to exclude excess cardiovascular risk at the time of first regulatory submission as requested by the FDA, EMA and other agencies^29,33,38^. The rationale for inclusion of HUA as a MACE component is driven by the advantage of increased numbers of events ascertained to ensure adequate statistical power for the assessment of cardiovascular safety and was prospectively planned. The secondary outcomes were MACE-3 (including cardiovascular death, MI and stroke), the composite outcome of MACE-3 or HHF as well as individual MACE components, including cardiovascular death, MI, stroke, HUA, HHF and all-cause death. Secondary outcomes also included the pooled analyses of trials with comparators with anticipated neutral effect on cardiovascular outcomes (insulins^18,21^) or placebo combined with best standard of care and the analysis of the trial with a selected high-risk cardiovascular population (SURPASS-4). Additional post hoc analyses included adjudicated outcome of coronary revascularization (urgent and non-urgent and surgical or percutaneous) and the first occurrence of any components of the composite outcome MACE-6 (including MACE-3 and all other adjudicated coronary outcomes (HUA, HHF and revascularization)) as well as subgroup analyses for race, country and SGLT-2i use at baseline.

### Statistical analyses

#### Meta-analysis

The primary aim of this cardiovascular safety meta-analysis, in accordance with FDA and EMA guidance^12–15^, was to demonstrate that tirzepatide was not associated with unacceptably high risk for cardiovascular events versus comparators, defined as an upper bound of the CI of the MACE-4 HR < 1.8.

The certainty of evidence obtained from this meta-analysis is high, and there is low basis to downgrade the quality of evidence according to GRADE methodology—based on bias, inconsistency, indirectness, imprecision or publication bias^39^. The intent-to-treat principle, high-quality execution of each of the prospectively randomized trials, the adjudication of endpoints by independent academic groups in a blinded manner and minimal missing data balanced between tirzepatide and comparator arms reduced potential bias (Supplementary Table 5). Plausible bias or indirectness due to the use of cardioprotective comparators, deemed to be minimal, could only bias cardiovascular event safety assessment against tirzepatide compared to control. In addition, the relatively shorter duration of up to 52 weeks of follow-up in clinical trials with low baseline cardiovascular risk compared to up to 104 weeks of follow-up in SURPASS-4 limits assessment for possible inconsistency.

A stratified Cox proportional hazards model was used with treatment (pooled tirzepatide groups and pooled comparator groups) as a fixed effect and stratified trial-level cardiovascular risk (SURPASS-4 and all other trials). This approach preserves the clustering of individual participant data when estimating parameters within strata^40^ and assumes (1) homogeneity of tirzepatide relative treatment effect on MACE regardless of patients’ baseline cardiovascular risk level; (2) homogeneity of tirzepatide treatment effect regardless of the comparator in each trial; and (3) proportional hazards of tirzepatide relative to comparator over time.

To account for different randomization ratios for pooled tirzepatide versus comparator between trials, adjusted estimates of means, standard deviations, event rates and percentages were obtained by weighting with inverse probability of randomization for treatment within stratum^41^. Cumulative incidence on time to first event was estimated using the adjusted Kaplan–Meier estimator weighting with inverse probability of randomization for treatment within stratum^42^. A stratified Cox proportional hazards regression model stratified by trial-level cardiovascular risk was also used for each subgroup analysis. The model contained treatment, the subgroup variable and the treatment-by-subgroup interaction term as fixed effects and trial-level cardiovascular risk stratification. All tests of interactions between treatment and subgroup were conducted at a two-sided alpha level of 0.10. Analyses of the following subgroups were pre-specified: sex, age (<65 years and ≥65 years) and baseline HbA1c (≤8.5% and >8.5%). Analyses of other subgroups were planned post hoc, including race (White or Non-White), country (US or non-US) and baseline SGLT-2i use (yes or no). The analyses included all randomized participants receiving at least one treatment dose (modified intent-to-treat (mITT) population), and all analyses were conducted using individual participant data in accordance with the intention-to-treat principle.

With these methods, assuming no difference in cardiovascular risk between tirzepatide and comparators, it was determined that 133 participants with MACE-4 would provide 90% power to exclude the upper limit of the 95% CI of 1.8 at two-sided alpha = 0.05. It was anticipated that approximately 110 of the 133 MACE-4 outcomers would be ascertained in the SURPASS-4 trial, which was designed as an event-driven trial continuing until 133 MACE-4 outcomes were confirmed, pooling results across all the relevant trials. Analyses were conducted using SAS Enterprise Guide version 7.1.

#### Interim analysis

An interim analysis was performed by an individual data monitoring committee, and the sponsor project members were kept blinded to the treatment assignments until individual trial data were locked. The purpose of the interim cardiovascular meta-analysis was to determine whether the pre-marketing cardiovascular safety requirement for regulatory submission was met. Assuming a 10% reduced cardiovascular risk with tirzepatide versus comparators, accrual of 100 participants with MACE-4 would provide 80% power to discharge an unacceptable increase in cardiovascular risk at approximately a two-sided significance level of 0.01. An interim meta-analysis was planned when at least 100 participants had experienced one component of MACE-4; when all global phase 3 trials, except SURPASS-4, were completed; and when regulatory-mandated long-term exposure requirements were met. To discharge an unacceptable increase in cardiovascular risk, the upper bound of the CI of the MACE-4 HR comparing pooled tirzepatide and pooled comparator less than 1.8 should be demonstrated. The alpha level for the interim analysis was to be calculated based on the fraction of 133 planned primary MACE-4 outcomes available at the interim analysis, with gamma of −6.6 guided by the Hwang–Shih–De Cani method^43^. If an unacceptable increase in cardiovascular risk was discharged at the interim analysis, close-out of the ongoing SURPASS-4 trial was to be initiated, with an anticipated close-out period of 3 months, during which accrual of additional MACE-4 was possible. Accordingly, the primary cardiovascular risk assessment of tirzepatide comprised the interim analysis, and the subsequent analyses using the final dataset after the completion of SURPASS-4 and SURPASS J-mono were conducted using a nominal significance level of 0.05 (95% CI). If an unacceptable increase in cardiovascular risk was not discharged at the interim analysis, the SURPASS-4 trial was to continue until accrual of 133 participants with MACE-4 occurred. In this setting, the primary cardiovascular risk assessment of tirzepatide was to be the analysis conducted with all available MACE-4 at the conclusion of SURPASS-4. The alpha level available for the final analysis was to be calculated based on the alpha spent at the interim analysis, the number of participants with MACE-4 at the interim analysis and the number of participants with MACE-4 at the final analysis.

#### Methods accounting for differences in randomization ratios and study-level cardiovascular risk

Due to the asymmetric randomization ratios and patient populations with different cardiovascular risk across the trials, the crude estimates simply aggregating the trials on each of the pooled tirzepatide group and the pooled comparator group could be misleading. Therefore, only adjusted estimates for each pooled treatment group were presented for summary statistics and event rate and cumulative incidence on time to first outcome. The adjustments were made by weighting with inverse probability of randomization for pooled treatment groups within stratum.

Within each stratum (SURPASS-4 only and all other trials), baseline cardiovascular risk factors were well balanced among the pooled treatment groups (Supplementary Tables 6 and 7), although patients’ background of cardiovascular risk was different between strata (for example, 86.9% of participants for SURPASS-4 only and 10.6% of participants for all other trials had history of cardiovascular disease). Thus, the crude estimates of percentage were different (for example, 28.9% of participants in pooled tirzepatide and 47.2% of participants in pooled comparator had history of cardiovascular disease) (Supplementary Table 8). After adjustment, the estimates were balanced among the pooled treatment groups (for example, 35.1% of participants in pooled tirzepatide and 34.4% of participants in pooled comparator had history of cardiovascular disease) (Supplementary Table 8). Adjusted estimates are the adequate values for comparing distribution among the pooled treatment groups.

### Reporting Summary

Further information on research design is available in the Nature Research Reporting Summary linked to this article.

## Online content

Any methods, additional references, Nature Research reporting summaries, source data, extended data, supplementary information, acknowledgements, peer review information; details of author contributions and competing interests; and statements of data and code availability are available at 10.1038/s41591-022-01707-4.

## Supplementary information

Supplementary Information Supplementary Tables 1–8

Reporting Summary

## Data Availability

Eli Lilly provides access to all individual participant data collected during the trial, after anonymization, with the exception of pharmacokinetic or genetic data. Data are available to request 6 months after the indication studied has been approved in the United States and the European Union and after primary publication acceptance, whichever is later. No expiration date of data requests is currently set once data are made available. Access is provided after a proposal has been approved by an independent review committee identified for this purpose and after receipt of a signed data sharing agreement. Data and documents, including the study protocol, statistical analysis plan, clinical study report and blank or annotated case report forms, will be provided in a secure data sharing environment. For details on submitting a request, see the instructions provided at www.vivli.org. Additional details of each trial assessed in these meta-analyses can be found at http://clinicaltrials.gov as NCT03131687 (phase 2), NCT03954834 (SURPASS-1), NCT03987919 (SURPASS-2), NCT03882970 (SURPASS-3), NCT03730662 (SURPASS-4), NCT04039503 (SURPASS-5) and NCT03861052 (SURPASS J-mono).
