# Supplementary material for: Tirzepatide cardiovascular event risk assessment: a pre-specified meta-analysis
Source: Nat Med. 2022 Feb 24;28(3):591–8. doi: 10.1038/s41591-022-01707-4 (PMC8938269; doi:10.1038/s41591-022-01707-4)
Supplement: Supplementary file 1 — Supplementary Tables 1–8 [file 41591_2022_1707_MOESM1_ESM.pdf]

nature portfolio
https://doi.org/10.1038/s41591-022-01707-4

Supplementary information

Tirzepatide cardiovascular event risk assessment: a pre-specified meta-analysis

In the format provided by the authors and unedited

ONLINE-ONLY SUPPLEMENTARY APPENDIX

Supplementary Table 1. Phase 2 and phase 3 tirzepatide trials included in the cardiovascular meta-analysis

Study	Overall N	Background Antihyperglycemic Therapy	Trial Design	Control/ Comparator	Treatment Duration[a]
**Phase 2 Trial**					
**I8F-MC-GPGB[7]**	316	± MET for ≥3 mo.	Randomized (TZP 4:PBO 1:DU 1), double-blind	PBO<br>DU 1.5 mg QW	26 wk
**Global Phase 3 Trials**					
**SURPASS-1[5]**	478	No OAM use for ≥3 mo.	Randomized (TZP 3:PBO 1), double-blind	PBO	40 wk
**SURPASS-2[6]**	1878	Stable MET (≥1500 mg/day) for ≥3 mo.	Randomized (TZP 3:SEMA 1), open-labeled[b]	SEMA 1.0 mg QW	40 wk
**SURPASS-3[35]**	1437	Insulin naïve, stable MET (≥1500 mg/day) ± SGLT-2i for ≥3 mo.	Randomized (TZP 3:iDEG 1), open-labeled	iDeg[c] QD	52 wk
**SURPASS-4[11]**	1995	Stable 1-3 OAMs (MET, SU, SGLT-2i) for ≥3 mo.	Randomized (TZP 3:iGlar 3), open-labeled	iGlar[d] QD	52-104 wk
**SURPASS-5[37]**	475	Insulin glargine ± MET for ≥3 mo.	Randomized (TZP 3:PBO 1), double-blind	PBO	40 wk
**Regional (Japan) Phase 3 Trial**					
**SURPASS J-mono[36]**	636	No OAM use for ≥2 mo.	Randomized (TZP 3:DU 1), double-blind	DU 0.75 mg QW	52 wk

[a]All trials included a 4-week follow-up period after the treatment period. [b]Tirzepatide doses were double-blinded. [c]Starting dose of 10 IU/day, titrated to fasting blood glucose <90 mg/dL, following a treat-to-target algorithm. [d]Starting dose of 10 IU/day, titrated to fasting blood glucose <100 mg/dL, following a treat-to-target algorithm. Abbreviations: DU = dulaglutide; iDeg = insulin degludec; iGlar = insulin glargine; MET = metformin; mo. = months; OAM = oral antihyperglycemic medication; PBO = placebo; QD = once daily; QW = once weekly; SU = sulfonylurea; SGLT-2i = sodium glucose co-transporter 2 inhibitor; TZP = tirzepatide; wk = weeks

Supplementary Table 2. Baseline cardiovascular risk assessment

Cardiovascular Risk Assessment	All Tirzepatide N=4199	All Comparator N=2064	Total N=6263
**History of cardiovascular disease**	1213 (35.1)	974 (34.4)	2187 (34.9)
**Prior myocardial infarction**	404 (11.8)	367 (12.5)	771 (12.3)
**Prior coronary revascularization procedure**	426 (12.4)	358 (12.4)	784 (12.5)
**Prior stroke**	170 (4.9)	138 (4.8)	308 (4.9)
**Prior hospitalization for unstable angina**	92 (2.7)	95 (3.2)	187 (3.0)
**Prior hospitalization for heart failure**	92 (2.7)	72 (2.5)	164 (2.6)
**Prior transient ischemic attack**	63 (1.8)	57 (2.0)	120 (1.9)
**Prior lower extremity arterial revascularization**	34 (1.0)	33 (1.2)	67 (1.1)
**Prior carotid revascularization**	21 (0.6)	18 (0.6)	39 (0.6)
**Hypertension**	3020 (73.8)	1607 (73.8)	4627 (73.9)
**Dyslipidemia**	2760 (67.7)	1477 (67.8)	4237 (67.7)
**Documented coronary artery disease**	611 (17.6)	508 (18.0)	1119 (17.9)
**Peripheral artery disease**	379 (11.3)	331 (11.5)	710 (11.3)
**Atrial fibrillation**	98 (2.6)	83 (3.2)	181 (2.9)
**First degree relatives diagnosed with coronary artery disease (males <55 years old, females <65 years old)**	367 (9.2)	168 (7.4)	535 (8.6)
**First degree relatives diagnosed with cerebrovascular disease**	294 (7.2)	164 (7.9)	458 (7.3)

Trials GPGB and SURPASS J-mono, where baseline cardiovascular risk assessment was not performed, were excluded. Data are n (strata size adjusted percentage). Data include all randomized participants who had the baseline cardiovascular risk assessment data recorded in the eCRF at the screening visit (Visit 1). Strata are defined as trial-level cardiovascular risk (SURPASS-4 forms one stratum, and all other trials form one stratum). Number of participants with non-missing data, used as denominator. Abbreviations: N = number of participants in population; n = number of participants in the specified category.

Supplementary Table 3. Baseline characteristics and cardiovascular risk assessment by trial

	SURPASS-1 N=478	SURPASS-2 N=1878	SURPASS-3 N=1437	SURPASS-4 N=1995	SURPASS-5 N=475
**Age, years (mean ± SD)**	54.1 ± 11.9	56.6 ± 10.4	57.4 ± 10.0	63.6 ± 8.6	60.6 ± 9.9
**Sex, female**	231 (48.3)	996 (53.0)	635 (44.2)	749 (37.5)	211 (44.4)
**T2D duration, years (mean ± SD)**	4.69 ± 5.38	8.62 ± 6.46	8.38 ± 6.24	11.78 ± 7.51	13.30 ± 7.31
**History of cardiovascular disease**	26 (5.4)	151 (8.0)	190 (13.2)	1733 (86.9)	87 (18.3)
**Prior myocardial infarction**	5 (1.0)	57 (3.0)	45 (3.1)	644 (32.3)	20 (4.2)
**Prior coronary revascularization procedure**	8 (1.7)	57 (3.0)	39 (2.7)	643 (32.2)	37 (7.8)
**Prior stroke**	2 (0.4)	23 (1.2)	25 (1.7)	240 (12.0)	18 (3.8)
**Prior hospitalization for unstable angina**	1 (0.2)	9 (0.5)	4 (0.3)	163 (8.2)	10 (2.1)
**Prior hospitalization for heart failure**	2 (0.4)	9 (0.5)	10 (0.7)	139 (7.0)	4 (0.8)
**Prior transient ischemic attack**	0	9 (0.5)	11 (0.8)	96 (4.8)	4 (0.8)
**Prior lower extremity arterial revascularization**	0	4 (0.2)	6 (0.4)	54 (2.7)	3 (0.6)
**Prior carotid revascularization**	1 (0.2)	1 (0.1)	2 (0.1)	34 (1.7)	1 (0.2)
**Hypertension**	252 (52.7)	1187 (63.2)	1024 (71.3)	1794 (89.9)	370 (77.9)
**Dyslipidemia**	202 (42.3)	1075 (57.2)	939 (65.3)	1666 (83.5)	355 (74.7)
**Documented coronary artery disease**	15 (3.1)	73 (3.9)	105 (7.3)	878 (44.0)	48 (10.1)
**Peripheral artery disease**	4 (0.8)	35 (1.9)	53 (3.7)	604 (30.3)	14 (2.9)
**Atrial fibrillation**	4 (0.8)	23 (1.2)	22 (1.5)	119 (6.0)	13 (2.7)
**First degree relatives diagnosed with coronary artery disease (males <55 years old, females <65 years old)**	21 (4.4)	145 (7.7)	84 (5.8)	238 (12.0)	47 (9.9)
**First degree relatives diagnosed with cerebrovascular disease**	32 (6.7)	130 (6.9)	82 (5.7)	168 (8.4)	46 (9.7)

Studies GPGB and SURPASS J-mono, where baseline cardiovascular risk assessment was not performed, were note presented. Data are n (percentage), unless otherwise noted. Number of participants with non-missing data, used as denominator. Abbreviations: N = number of participants in population; n = number of participants in the specified category; SD = standard deviation.

Supplementary Table 4. Summary of adjudication-confirmed MACE-4 by trial

Trial	Pooled Treatments n/N (%) [Event Rate]
**GPGB**	2/316 (0.6) [1.13]
**SURPASS-1**	1/478 (0.2) [0.26]
**SURPASS-2**	14/1878 (0.7) [0.89]
**SURPASS-3**	10/1437 (0.7) [0.67]
**SURPASS-4**	109 /1995 (5.5) [3.52]
**SURPASS-5**	3/475 (0.6) [0.77]
**SURPASS J-mono**	3/636 (0.5) [0.45]

Event rate: number of patients experiencing MACE-4 per 100 person-years. Abbreviations; N = number of participants in population; n = number of patients who experienced at least one component of MACE-4.

Supplementary Table 5. Assessment of bias for individual trials included in the meta-analysis

Trial	Risk of Bias Domains					
	D1	D2	D3	D4	D5	Overall
**GPGB**	+	+	+	+	+	+
**SURPASS-1**	+	+	+	+	+	+
**SURPASS-2**	+	+	+	+	+	+
**SURPASS-3**	+	+	+	+	+	+
**SURPASS-4**	+	+	+	+	+	+
**SURPASS-5**	+	+	+	+	+	+
**SURPASS J-mono**	+	+	+	+	+	+

Judgement:
+ Low

Domains:
D1: Bias arising from the randomization process.
D2: Bias due to deviations from intended intervention.
D3: Bias due to missing outcome data.
D4: Bias in measurement of the outcome.
D5: Bias in section of the reported result.

Supplementary Table 6. Baseline characteristics of cardiovascular risk – SURPASS-4 only

Cardiovascular Risk Assessment	All Tirzepatide N=995	All Comparator N=1000	Total N=1995
**History of cardiovascular disease**	864 (86.8)	869 (86.9)	1733 (86.9)
**Prior myocardial infarction**	302 (30.4)	342 (34.2)	644 (32.3)
**Prior coronary revascularization procedure**	315 (31.7)	328 (32.8)	643 (32.2)
**Prior stroke**	116 (11.7)	124 (12.4)	240 (12.0)
**Prior hospitalization for unstable angina**	73 (7.3)	90 (9.0)	163 (8.2)
**Prior hospitalization for heart failure**	72 (7.2)	67 (6.7)	139 (7.0)
**Prior transient ischemic attack**	45 (4.5)	51 (5.1)	96 (4.8)
**Prior lower extremity arterial revascularization**	25 (2.5)	29 (2.9)	54 (2.7)
**Prior carotid revascularization**	18 (1.8)	16 (1.6)	34 (1.7)
**Hypertension**	888 (89.2)	906 (90.6)	1794 (89.9)
**Dyslipidemia**	835 (83.9)	831 (83.1)	1666 (83.5)
**Documented coronary artery disease**	425 (42.7)	453 (45.3)	878 (44.0)
**Peripheral artery disease**	304 (30.6)	300 (30.0)	604 (30.3)
**Atrial fibrillation**	52 (5.2)	67 (6.7)	119 (6.0)
**First degree relatives diagnosed with coronary artery disease (males <55 years old, females <65 years old)**	133 (13.4)	105 (10.5)	238 (12.0)
**First degree relatives diagnosed with cerebrovascular disease**	86 (8.7)	82 (8.2)	168 (8.4)

Data are n (crude percentage). Data include all randomized participants who had the baseline cardiovascular risk assessment data recorded in the eCRF at the screening visit (Visit 1). Number of participants with non-missing data, used as denominator. Abbreviations: N = number of participants in population; n = number of participants in the specified category.

Supplementary Table 7. Baseline characteristics cardiovascular risk assessment – all studies except SURPASS-4

Cardiovascular Risk Assessment	All Tirzepatide N=3204	All Comparator N=1064	Total N=4268
**History of cardiovascular disease**	349 (10.9)	105 (9.9)	454 (10.6)
**Prior myocardial infarction**	102 (3.2)	25 (2.3)	127 (3.0)
**Prior coronary revascularization procedure**	111 (3.5)	30 (2.8)	141 (3.3)
**Prior stroke**	54 (1.7)	14 (1.3)	68 (1.6)
**Prior hospitalization for unstable angina**	19 (0.6)	5 (0.5)	24 (0.6)
**Prior hospitalization for heart failure**	20 (0.6)	5 (0.5)	25 (0.6)
**Prior transient ischemic attack**	18 (0.6)	6 (0.6)	24 (0.6)
**Prior lower extremity arterial revascularization**	9 (0.3)	4 (0.4)	13 (0.3)
**Prior carotid revascularization**	3 (0.1)	2 (0.2)	5 (0.1)
**Hypertension**	2132 (66.5)	701 (65.9)	2833 (66.4)
**Dyslipidemia**	1925 (60.1)	646 (60.7)	2571 (60.2)
**Documented coronary artery disease**	186 (5.8)	55 (5.2)	241 (5.6)
**Peripheral artery disease**	75 (2.3)	31 (2.9)	106 (2.5)
**Atrial fibrillation**	46 (1.6)	16 (1.5)	62 (1.5)
**First degree relatives diagnosed with coronary artery disease (males <55 years old, females <65 years old)**	234 (7.3)	63 (5.9)	297 (7.0)
**First degree relatives diagnosed with cerebrovascular disease**	208 (6.5)	82 (7.7)	290 (6.8)

Trials GPGB and SURPASS J-mono, where baseline cardiovascular risk assessment was not performed, were excluded. Data are n (crude percentage). Data include all randomized participants who had the baseline cardiovascular risk assessment data recorded in the eCRF at the screening visit (Visit 1). Number of participants with non-missing data, used as denominator. Abbreviations: N = number of participants in population; n = number of participants in the specified category.

Supplementary Table 8. Unadjusted and adjusted baseline cardiovascular risk assessment – all patients

Cardiovascular Risk Assessment	All Tirzepatide N=4199	All Comparator N=2064	Total N=6263
**History of cardiovascular disease**	1213 (28.9) [35.1]	974 (47.2) [34.4]	2187 (34.9)
**Prior myocardial infarction**	404 (9.6) [11.8]	367 (17.8) [12.5]	771 (12.3)
**Prior coronary revascularization procedure**	426 (10.1) [12.4]	358 (17.3) [12.4]	784 (12.5)
**Prior stroke**	170 (4.0) [4.9]	138 (6.7) [4.8]	308 (4.9)
**Prior hospitalization for unstable angina**	92 (2.2) [2.7]	95 (4.6) [3.2]	187 (3.0)
**Prior hospitalization for heart failure**	92 (2.2) [2.7]	72 (3.5) [2.5]	164 (2.6)
**Prior transient ischemic attack**	63 (1.5) [1.8]	57 (2.8) [2.0]	120 (1.9)
**Prior lower extremity arterial revascularization**	34 (0.8) [1.0]	33 (1.6) [1.2]	67 (1.1)
**Prior carotid revascularization**	21 (0.5) [0.6]	18 (0.9) [0.6]	39 (0.6)
**Hypertension**	3020 (71.9) [73.8]	1607 (77.9) [73.8]	4627 (73.9)
**Dyslipidemia**	2760 (65.7) [67.7]	1477 (71.6) [67.8]	4237 (67.7)
**Documented coronary artery disease**	611 (14.6) [17.6]	508 (24.6) [18.0]	1119 (17.9)
**Peripheral artery disease**	379 (9.0) [11.3]	331 (16.0) [11.5]	710 (11.3)
**Atrial fibrillation**	98 (2.3) [2.6]	83 (4.0) [3.2]	181 (2.9)
**First degree relatives diagnosed with coronary artery disease (males <55 years old, females <65 years old)**	367 (8.8) [9.2]	168 (8.2) [7.4]	535 (8.6)
**First degree relatives diagnosed with cerebrovascular disease**	294 (7.0) [7.2]	164 (8.0) [7.9]	458 (7.3)

Trials GPGB and SURPASS J-mono, where baseline cardiovascular risk assessment was not performed, were excluded. Data are n (crude percentage) [strata size adjusted estimate of percentage]. Data include all randomized participants who had the baseline cardiovascular risk assessment data recorded in the eCRF at the screening visit (Visit 1). Strata are defined as trial-level cardiovascular risk (SURPASS-4 forms one stratum, and all other trials form one stratum). Number of participants with non-missing data, used as denominator. Abbreviations: N = number of participants in population; n = number of participants in the specified category.
