# Supplementary material for: Tirzepatide cardiovascular event risk assessment: a pre-specified meta-analysis
Source: Nat Med. 2022 Feb 24;28(3):591–8. doi: 10.1038/s41591-022-01707-4 (PMC8938269; doi:10.1038/s41591-022-01707-4)
Supplement: Supplementary file 2 — Reporting Summary [file 41591_2022_1707_MOESM2_ESM.pdf]

nature research

Corresponding author(s): Naveed Sattar and Sophia Zoungas

Last updated by author(s): January 10, 2022

Reporting Summary

Nature Research wishes to improve the reproducibility of the work that we publish. This form provides structure for consistency and transparency in reporting. For further information on Nature Research policies, see our Editorial Policies and the Editorial Policy Checklist.

Statistics

For all statistical analyses, confirm that the following items are present in the figure legend, table legend, main text, or Methods section.

n/a	Confirmed	
☐	☒	The exact sample size (*n*) for each experimental group/condition, given as a discrete number and unit of measurement
☐	☒	A statement on whether measurements were taken from distinct samples or whether the same sample was measured repeatedly
☐	☒	The statistical test(s) used AND whether they are one- or two-sided<br>*Only common tests should be described solely by name; describe more complex techniques in the Methods section.*
☐	☒	A description of all covariates tested
☐	☒	A description of any assumptions or corrections, such as tests of normality and adjustment for multiple comparisons
☐	☒	A full description of the statistical parameters including central tendency (e.g. means) or other basic estimates (e.g. regression coefficient) AND variation (e.g. standard deviation) or associated estimates of uncertainty (e.g. confidence intervals)
☐	☒	For null hypothesis testing, the test statistic (e.g. *F*, *t*, *r*) with confidence intervals, effect sizes, degrees of freedom and *P* value noted<br>*Give P values as exact values whenever suitable.*
☒	☐	For Bayesian analysis, information on the choice of priors and Markov chain Monte Carlo settings
☐	☒	For hierarchical and complex designs, identification of the appropriate level for tests and full reporting of outcomes
☒	☐	Estimates of effect sizes (e.g. Cohen's *d*, Pearson's *r*), indicating how they were calculated

Our web collection on statistics for biologists contains articles on many of the points above.

Software and code

Policy information about availability of computer code

Data collection	No software was used for data collection. Analyses were conducted using SAS Enterprise Guide V7.1.
Data analysis	Analyses were conducted using SAS Enterprise Guide V7.1.

For manuscripts utilizing custom algorithms or software that are central to the research but not yet described in published literature, software must be made available to editors and reviewers. We strongly encourage code deposition in a community repository (e.g. GitHub). See the Nature Research guidelines for submitting code & software for further information.

Data

Policy information about availability of data

All manuscripts must include a data availability statement. This statement should provide the following information, where applicable:

- Accession codes, unique identifiers, or web links for publicly available datasets
- A list of figures that have associated raw data
- A description of any restrictions on data availability

Lilly provides access to all individual participant data collected during the trial, after anonymization, with the exception of pharmacokinetic or genetic data. Data are available to request 6 months after the indication studied has been approved in the US and EU and after primary publication acceptance, whichever is later. No expiration date of data requests is currently set once data are made available. Access is provided after a proposal has been approved by an independent review committee identified for this purpose and after receipt of a signed data sharing agreement. Data and documents, including the study protocol, statistical analysis plan, clinical study report, blank or annotated case report forms, will be provided in a secure data sharing environment. For details on submitting a request, see the instructions provided at www.vivli.org. Additional details of each trial assessed in these meta-analyses can be found at http://clinicaltrials.gov as NCT03131687 (Phase 2), NCT03954834 (SURPASS-1), NCT03987919 (SURPASS-2), NCT03882970 (SURPASS-3), NCT03730662 (SURPASS-4), NCT04039503 (SURPASS-5) and NCT03861052 (SURPASS J-mono).

Field-specific reporting

Please select the one below that is the best fit for your research. If you are not sure, read the appropriate sections before making your selection.

☒ Life sciences ☐ Behavioural & social sciences ☐ Ecological, evolutionary & environmental sciences

For a reference copy of the document with all sections, see nature.com/documents/nr-reporting-summary-flat.pdf

Life sciences study design

All studies must disclose on these points even when the disclosure is negative.

Sample size	Determination of sample size was reported in each individual trial included in this meta-analysis. For this meta-analysis and under the assumption of no excess CV risk with tirzepatide compared with comparators, the sample size was determined for selected combinations of the number of MACE-4 from SURPASS-4 and the number of MACE-4 from other Ph 3 trials combined, leading to statistical power of at least 90% to demonstrate upper bound of 95% CI for HR<1.8.
Data exclusions	Two studies were excluded: one Phase 2 trial with 111 participants and 12 weeks duration and one uncontrolled Phase 3 safety trial conducted in Japan. Trial durations ranged from 26-104 weeks (Supplementary Table 1). Individual data from participants randomized to tirzepatide (1 mg, 5 mg, 10 mg or 15 mg) and randomized to placebo or active comparator (insulin degludec, insulin glargine, semaglutide 1 mg, or dulaglutide [1.5 mg or 0.75 mg]) were pooled. Furthermore, this CV meta-analysis included tirzepatide clinical trials that excluded participants with recent unstable CV disease (e.g., the New York Heart Association Class IV heart failure and recent CV events).
Replication	Results of these meta-analysis are based on randomized clinical trials conducted in accordance with the Declaration of Helsinki and Good Clinical Practice guidelines with CV outcomes adjudicated by an independent clinical endpoint committee in a blinded fashion.
Randomization	Participants were randomly assigned 2:1 to tirzepatide (1 mg, 5 mg, 10 mg or 15 mg) or comparator (placebo or dulaglutide 1.5 mg) in the Phase 2 trial, 3:1 to tirzepatide (5 mg, 10 mg or 15 mg) or comparators (placebo, semaglutide 1 mg, insulin degludec or dulaglutide 0.75 mg) in the Phase 3 trials other than SURPASS-4, and 1:1 to tirzepatide (5 mg, 10 mg, or 15 mg) or insulin glargine in SURPASS-4.
Blinding	Blinding was reported in each individual trial included in this meta-analysis and in Supplementary Table 1. In summary, Ph 2 GPGB, SURPASS-1, SURPASS-5 and SURPASS J-mono were double blind and investigators were blinded to group allocation during data collection and analysis. SURPASS-2, -3 and -4 were open-labeled. SURPASS-2 was an active-controlled study with semaglutide 1 mg and could not be blinded because of differences in devices. SURPASS-3 and -4 were active-controlled studies with insulin and could not be blinded because of differences in dosing schedules and devices. All studies were randomized.

Reporting for specific materials, systems and methods

We require information from authors about some types of materials, experimental systems and methods used in many studies. Here, indicate whether each material, system or method listed is relevant to your study. If you are not sure if a list item applies to your research, read the appropriate section before selecting a response.

Materials & experimental systems

n/a	Involved in the study	
☒	☐	Antibodies
☒	☐	Eukaryotic cell lines
☒	☐	Palaeontology and archaeology
☒	☐	Animals and other organisms
☐	☒	Human research participants
☐	☒	Clinical data
☒	☐	Dual use research of concern

Methods

n/a	Involved in the study	
☒	☐	ChIP-seq
☒	☐	Flow cytometry
☒	☐	MRI-based neuroimaging

Human research participants

Policy information about studies involving human research participants

Population characteristics	The trials included individuals aged ≥18 years with T2D inadequately controlled with diet and exercise ± metformin, with a baseline HbA1c ranging from 7.0-10.5%, and BMI ≥23 kg/m2, depending on the trial. Importantly, SURPASS-4 included a high CV risk population and was designed to contribute the majority (i.e., approximately 80%) of the MACE-4 endpoints for evaluating CV safety, as compared with the other studies. The baseline demographic characteristics were balanced across the pooled tirzepatide and pooled comparator group. Overall, participants had a mean a baseline HbA1c 8.3%, a mean diabetes duration of 9.3 years, and a mean BMI of 32.8 kg/m2, a mean age of 59 years and 43% were female.
Recruitment	Recruitment was based on inclusion and exclusion criteria for adults with type 2 diabetes.

Ethics oversight	Each trial was prospectively registered at Clinicaltrials.gov, had Institutional Review Board approval for each participating center, and was conducted in accordance with the Declaration of Helsinki. All participants provided written informed consent for trial participation.

Note that full information on the approval of the study protocol must also be provided in the manuscript.

Clinical data

Policy information about clinical studies

All manuscripts should comply with the ICMJE guidelines for publication of clinical research and a completed CONSORT checklist must be included with all submissions.

Clinical trial registration	Additional details of trials assessed in these meta-analyses can be found at http://clinicaltrials.gov as NCT03131687 (Phase 2), NCT03954834 (SURPASS-1), NCT03987919 (SURPASS-2), NCT03882970 (SURPASS-3), NCT03730662 (SURPASS-4), NCT04039503 (SURPASS-5) and NCT03861052 (SURPASS J-mono).
Study protocol	Study protocols of all published are trials are provided in the supplemental appendix of the primary manuscript submissions to NEJM (SURPASS-2) and Lancet (SURPASS-1, SURPASS-3, SURPASS-4 and Ph2 GPGB) Protocols of (SURPASS-5 and J-mono) will be available as soon as the corresponding primary manuscripts are published.
Data collection	For the Ph 2 GPGB trial (NCT03131687) data were collected at 47 sites (medical and clinical research centres) in Poland, Puerto Rico, Slovakia, and USA during May 2017 - August 2018. For the SURPASS-1 trial (NCT03954834), data were collected at 52 medical research centers and hospitals in India, Japan, Mexico and the USA during June 2019- October 2020. For the SURPASS-2 trial (NCT03987919), data were collected in 28 sites in the USA, Argentina, Australia, Brazil, Canada, Israel, Mexico, and UK during July 2019-February 2021, For the SURPASS-3 trial (NCT03882970), data were collected in 121 sites in Argentina, Austria, Greece, Hungary, Italy, Poland, Romania, South Korea, Spain, Taiwan, Ukraine, and the United States during April 2019-January 2021. For the SURPASS-4 trial (NCT03730662), data were collected in 187 sites in Argentina, Australia, Brazil, Canada, Greece, Israel, Mexico, Poland, Romania, Russia, Slovakia, Spain, Taiwan, and the USA during November 2018-April 2021. For the SURPASS-5 trial (NCT04039503), data were collected in one center in the USA during August 2019 - January 2021.For the SURPASS J-mono trial (NCT03861052), data were collected in multiple sites in Japan during May 2017 - March 2021. Data were collected using the Electronic Data Capture system.
Outcomes	The primary outcome was time to first occurrence of CEC-confirmed MACE-4 (including CV death, MI, stroke, and HUA). MACE-4 has been used in meta-analyses for several new diabetes medications to exclude excess CV risk at the time of first regulatory submission as requested by the FDA, EMA and other agencies. The rationale for inclusion of hospitalization for unstable angina as a MACE component is driven by the advantage of increased numbers of events ascertained to ensure adequate statistical power for the assessment of CV safety and was prospectively planned. The secondary outcomes were MACE-3 (including CV death, MI and stroke), the composite outcome of MACE-3 or HHF, as well as individual MACE components, including CV death, MI, stroke, HUA, HHF, and all-cause death. Secondary outcomes also included the pooled analyses of trials with comparators with anticipated neutral effect on CV outcomes (insulins) or placebo combined with best standard of care) and the analysis of the trial with a selected high risk CV population (SURPASS-4). Additional post-hoc analyses included adjudicated outcome of coronary revascularization (urgent and non-urgent; surgical or percutaneous) and the first occurrence of any components of the composite outcome MACE-6 (including MACE-3 and all other adjudicated coronary outcomes [HUA, HHF and revascularization]), as well as subgroup analyses for race, country and SGLT-2i use at baseline.<br>The primary aim of this CV safety meta-analysis, in accordance with the FDA and EMA guidance, was to demonstrate that tirzepatide was not associated with unacceptably high risk for CV events versus comparators, defined as an upper bound of the confidence interval (CI) of the 4-component major adverse CV event hazard ratio (MACE-4 HR) <1.8.<br>The certainty of evidence obtained from this meta-analysis is high and there is low basis to downgrade quality of evidence according to GRADE methodology: based on bias, inconsistency, indirectness, imprecision, or publication bias. The intent-to-treat principle, high quality execution of each of the prospectively randomized trials, the adjudication of endpoints by independent academic groups in a blinded manner, and minimal missing data balanced between tirzepatide and comparator arms reduced potential bias (Table S5). Plausible bias or indirectness due to the use of cardioprotective comparators, deemed to be minimal, could only bias CV event safety assessment against tirzepatide compared to control. In addition, the relatively shorter duration of up to 52 weeks follow-up in clinical trials with low baseline CV risk compared to up to 104 weeks of follow-up in SURPASS-4 limits assessment for possible inconsistency.<br>A stratified Cox proportional hazards model was used with treatment (pooled tirzepatide groups, pooled comparator groups) as a fixed effect and stratified trial-level CV risk (SURPASS-4, all other trials). This approach preserves the clustering of individual participant data when estimating parameters within strata. and assumes a) homogeneity of tirzepatide relative treatment effect on MACE regardless of patient's baseline CV risk level; b) homogeneity of tirzepatide treatment effect regardless of the comparator in each trial; and c) proportional hazards of tirzepatide relative to comparator over time.<br>To account for different randomization ratios for pooled tirzepatide versus comparator between trials, adjusted estimates of means, standard deviations (SD), event rates, and percentages were obtained by weighting with inverse probability of randomization for treatment within stratum. Cumulative incidence on time to first event was estimated using the adjusted Kaplan-Meier estimator weighting with inverse probability of randomization for treatment within stratum. A stratified Cox proportional hazards regression model stratified by trial-level CV risk was also used for each subgroup analysis. The model contained treatment, the subgroup variable, and the treatment-by-subgroup interaction term as fixed effects and trial-level CV risk stratification. All tests of interactions between treatment and subgroup were conducted at a 2-sided alpha level of 0.10. Analyses of the following subgroups were pre-specified: sex, age (<65 years, ≥65 years), and baseline HbA1c (≤8.5%, >8.5%). Analyses of other subgroups were planned post hoc, including race (White, Non-White), country (US, non-US), and baseline SGLT-2i use (yes, no). The analyses included all randomized participants receiving at least one treatment dose (modified intent-to-treat population) and all analyses were conducted using individual participant data in accord with the intention to treat principle.
